# Supplementary material for: MetaQTL: a package of new computational methods for the meta-analysis of QTL mapping experiments
Source: BMC Bioinformatics. 2007 Feb 8;8:49. doi: 10.1186/1471-2105-8-49 (PMC1808479; doi:10.1186/1471-2105-8-49)
Supplement: Additional File 7 — MetaQTL Package : jar file and tutorial. This Zip archive contains both the MetaQTL JAR file and the files of the tutorial. [file 1471-2105-8-49-S7.zip › org.inra.metaqtl/doc/org/thalia/bio/entity/factory/MapChartFactory.html]

MapChartFactory


|  |  |  |
| --- | --- | --- |
| **Overview** \| **Package** \| **Class** \| **Use** \| **Tree** \| **Deprecated** \| **Index** \| **Help** | |  |
| **PREV CLASS**   **NEXT CLASS** | **FRAMES**    **NO FRAMES**     **All Classes** |  |
| SUMMARY: NESTED \| FIELD \| CONSTR \| METHOD | DETAIL: FIELD \| CONSTR \| METHOD |  |


---


## org.thalia.bio.entity.factory Class MapChartFactory

```
java.lang.Object
  org.thalia.bio.entity.factory.BioEntityFactory
      org.thalia.bio.entity.factory.MapChartFactory
```

---

``` public class MapChartFactory extends BioEntityFactory ```

General comments must be added here.

**Author:**
:   Julien Cornouiller
    Additions must be added here.

---

**Constructor Summary**

`MapChartFactory()`


| **Method Summary** | |
| --- | --- |
| `IBioEntity` | `load(java.io.InputStream stream)`             TODO (non-Javadoc) |
| `IBioEntity` | `load(java.io.Reader reader)`             TODO (non-Javadoc) |
| `void` | `unload(IBioEntity obj, java.io.OutputStream stream)`             TODO (non-Javadoc) |
| `void` | `unload(IBioEntity obj, java.io.Writer w)`             TODO (non-Javadoc) |

**Methods inherited from class java.lang.Object**

`clone, equals, finalize, getClass, hashCode, notify, notifyAll, toString, wait, wait, wait`

**Constructor Detail**

### MapChartFactory

```
public MapChartFactory()
```


**Method Detail**

### load

```
public IBioEntity load(java.io.Reader reader)
                throws java.io.IOException
```

:   TODO (non-Javadoc)

    :   **Specified by:**: `load` in class `BioEntityFactory`
    :   **Throws:**: `java.io.IOException` **See Also:**: `org.thalia.core.runtime.ITiedPluginFactory#load(java.io.Reader)`

---


### load

```
public IBioEntity load(java.io.InputStream stream)
                throws java.io.IOException
```

:   TODO (non-Javadoc)

    :   **Specified by:**: `load` in class `BioEntityFactory`
    :   **Throws:**: `java.io.IOException` **See Also:**: `org.thalia.core.runtime.ITiedPluginFactory#load(java.io.InputStream)`

---


### unload

```
public void unload(IBioEntity obj,
                   java.io.Writer w)
            throws java.io.IOException
```

:   TODO (non-Javadoc)

    :   **Specified by:**: `unload` in class `BioEntityFactory`
    :   **Throws:**: `java.io.IOException` **See Also:**: `org.thalia.core.runtime.ITiedPluginFactory#unload(java.lang.Object, java.io.Writer)`

---


### unload

```
public void unload(IBioEntity obj,
                   java.io.OutputStream stream)
            throws java.io.IOException
```

:   TODO (non-Javadoc)

    :   **Specified by:**: `unload` in class `BioEntityFactory`
    :   **Throws:**: `java.io.IOException` **See Also:**: `org.thalia.core.runtime.ITiedPluginFactory#unload(java.lang.Object, java.io.OutputStream)`


---


|  |  |  |
| --- | --- | --- |
| **Overview** \| **Package** \| **Class** \| **Use** \| **Tree** \| **Deprecated** \| **Index** \| **Help** | |  |
| **PREV CLASS**   **NEXT CLASS** | **FRAMES**    **NO FRAMES**     **All Classes** |  |
| SUMMARY: NESTED \| FIELD \| CONSTR \| METHOD | DETAIL: FIELD \| CONSTR \| METHOD |  |


---
